# Framework of behavioral indicators for outcome evaluation of TB health promotion: a Delphi study of TB suspects and Tb patients

**DOI:** 10.1186/1471-2334-14-268

**Published:** 2014-05-16

**Authors:** Ying Li, John Ehiri, Daiyu Hu, Yanqi Zhang, Qingya Wang, Shun Zhang, Jia Cao

**Affiliations:** 1 Department of Social Medicine and Health Service Management, Third Military Medical University, No.30 Gaotanyan Road, Shapingba district, Chongqing 400038, China; 2 Division of Health Promotion Sciences/Global Health Institute, Mel & Enid Zuckerman College of Public Health, University of Arizona, Tucson, Arizona, USA; 3 Chongqing Institute of TB Prevention and Treatment, Jiulongpo district, Chongqing, China; 4 Department of Health statistics, College of Preventive Medicine, Third Military Medical University, Chongqing, China; 5 College of Preventive Medicine, Key Lab of Medical Protection for Electromagnetic Radiation, Ministry of Education of China, Third Military Medical University, Chongqing, China

**Keywords:** Health promotion, Outcome evaluation, TB suspect, TB patient, Indicator

## Abstract

**Background:**

Health promotion for prevention and control of Tuberculosis (TB) is implemented worldwide because of its importance, but few reports have evaluated its impact on behavior due to a lack of standard outcome indicators. The objective of this study was to establish a framework of behavioral indicators for outcome evaluation of TB health promotion among TB suspects and patients.

**Methods:**

A two-round modified Delphi method involving sixteen TB control experts was used to establish a framework of behavioral indicators for outcome evaluation of TB health promotion targeted at TB suspects and patients.

**Results:**

Sixteen of seventeen invited experts in TB control (authority score of 0.91 on a 1.0 scale) participated in round 1 survey. All sixteen experts also participated in a second round survey. After two rounds of surveys and several iterations among the experts, there was consensus on a framework of indicators for measuring outcomes of TB health promotion for TB suspects and patients. For TB suspects, the experts reached consensus on 2 domains (“Healthcare seeking behavior” and “Transmission prevention”), 3 subdomains (“Seeking care after onset of TB symptoms”, “Pathways of seeking care” and “Interpersonal contact etiquette”), and 8 indicators (including among others, “Length of patient delay”). For TB patients, consensus was reached on 3 domains (“Adherence to treatment”, “Healthy lifestyle” and “Transmission prevention”), 8 subdomains (including among others, “Adherence to their medication”), and 14 indicators (including “Percentage of patients who adhered to their medication”). Operational definitions and data sources were provided for each indicator.

**Conclusions:**

The findings of this study provide the basis for debate among international experts on a framework for achieving global consensus on outcome indicators for TB health promotion interventions targeted at TB patients and suspects. Such consensus will help to increase effectiveness of TB health promotion, while ensuring international comparability of outcome data.

## Background

Although significant progress has been made in global Tuberculosis (TB) control over the past decade, the disease remains an abiding global health challenge [1]. TB is transmitted primarily through the airborne route, and key control measures are early diagnosis and prompt treatment of individuals with active disease, and identification and treatment of latent infections [2]. However, delays in diagnosis and treatment, and treatment failures resulting from low adherence are common in many low and middle income countries with high TB burdens [1,3-10]. There is considerable literature on individual and health systems factors that influence patient, diagnostic, and treatment delays [8,11]. Factors that contribute to poor adherence to TB treatment and prevention, and barrier in multi-drug resistant TB (MDR-TB) treatment have also been widely documented [12-14]. Many of the factors that contribute to TB proliferation are modifiable through health promotion, yet, TB continues to present significant threats to health in high burden countries, and the emergence of multi-drug resistance continues to increase globally [1]. Health Promotion is defined as the process of enabling people to increase control over, and to improve their health [15]. TB health promotion includes individual empowerment, community empowerment, health systems strengthening, interagency partnerships, and intersectional collaboration [16]. For TB suspects and TB patients, health promotion would play a key role in improving TB knowledge and awareness, health seeking behavior, treatment adherence, and thus, treatment outcome [17-19].

Given the public health significance of TB, health promotion interventions to prevent and control the disease have been conducted, and continue to be conducted globally. In some cases, these interventions are aimed at strengthening people’s understanding of the disease and how to respond to symptoms [20-24]. Such interventions have also been used to reduce TB stigma [18,19], a major cause of TB diagnostic and treatment delay and poor adherence to treatment [11,17,21,25,26]. Overall, these TB health promotion activities have sought to influence behavior change and encourage adherence to treatment [17] through multifaceted package of interventions [21,25,26]. Some countries, including China [27,28] and New Zealand [16], have issued special guidelines for TB health promotion that specify TB health promotion activities targeting behavioral interventions for different populations groups [17]. Unfortunately, these guidelines lack appropriate indicators for assessment of outcomes. In the New Zealand guideline for example, measures of quality and effectiveness of TB health promotion and education are deficient [16]. In China, the 2008 *Guideline on Enforcement of Chinese Tuberculosis Control Program* describes only two evaluation indicators (rate of completion of health promotion activities, and TB core knowledge of target participants) [28]. Unfortunately, knowledge alone often does not translate to actual behavior changes [29-31].

Evaluation of health programs is important to assess the extent to which programs meet stated objectives [32]. Evaluation usually includes assessment of inputs, processes, outputs, outcomes, and impacts [32-34]. Outcome evaluation assesses the short and medium term effects of an intervention (e.g., behavior change or changes in health status) and correlates them with the program's objectives [34]. Having appropriate indicators for ascertaining achievement of program objectives is key to meaningful evaluation, and such indicators should be identified and determined in the initial stages of program planning [35,36]. Although other major diseases of global significance (including HIV/AIDS) have universally accepted framework of indicators for assessing the impact of health promotion activities [35,37], the global TB control effort has no such framework of indicators for its health promotion.

The only available framework for monitoring and evaluating national TB control programs was that issued by the World Health Organization in 2004 [38-40]. Unfortunately, this framework only covers outcome of detection and treatment, but neglects other important behavioral changes that may be associated with TB health promotion interventions. To facilitate the development of a universal framework of outcome indicators to guide TB health promotion interventions in countries with high TB burden, we conducted a study to establish behavioral indicators for outcome evaluation of individual level TB health promotion, using the Delphi method [41].

## Methods

We used the Delphi method to establish the framework of indicators for individual level TB health promotion. The Delphi method is a consensus technique that collects expert opinions through several rounds of surveys or interviews. It has 4 distinguishing features: anonymity, iteration, controlled feedback, and statistical group response (expression of the degree of consensus within a group) [42-45]. From various Delphi methodologies [41], we chose the Modified Delphi method, which includes two quantitative rounds of surveys that were conducted from May to October 2012.

### Selection of Delphi experts

The purposive sampling technique [46] was used to select informed individuals to serve in a panel of experts for the Delphi process. Expertise and eligibility were determined using the following criteria:

1) Policy maker at national or provincial level who had been working for at least 5 years in TB diagnosis, treatment, or prevention.

2) Senior TB professional with in-depth knowledgeable and experienced in TB control (including physicians directly involved in TB diagnosis and treatment).

3) National coverage – representation from eastern, western, northern, and southern China to ensure national generalizability of results.

A total of 17 TB control experts from national and provincial level TB control facilities participated in the Delphi process. During recruitment, potential experts were approached (initially via e-mail and later by telephone) and provided with detailed explanation of the study and its objectives. They were then asked if they would be interested in volunteering to participate. Those who expressed interest were asked to read the informed consent form, and were assured of confidentiality. They were sent an informed consent form to review, sign and return in an addressed stamped envelope as a conformation of their voluntary participation in the study. Ethical approval for the study was obtained from the Health Research Ethics Board of Third Military Medical University, China.

### Instrument

The instrument for the Delphi surveys consisted of four parts:

1) instructions for the Delphi survey, used to introduce the study Domains and to clarify how to complete the survey;

2) questionnaire (main instrument) with indicators for evaluating the impact of individual level TB health promotion;

3) information about experts’ authority in the field of TB control (familiarization with indicators and judgment criteria for the indicator), and

4) general information about the experts, such as (age, professional title, position, education, major, duration of work in the field of TB control).

**Figure 1:**
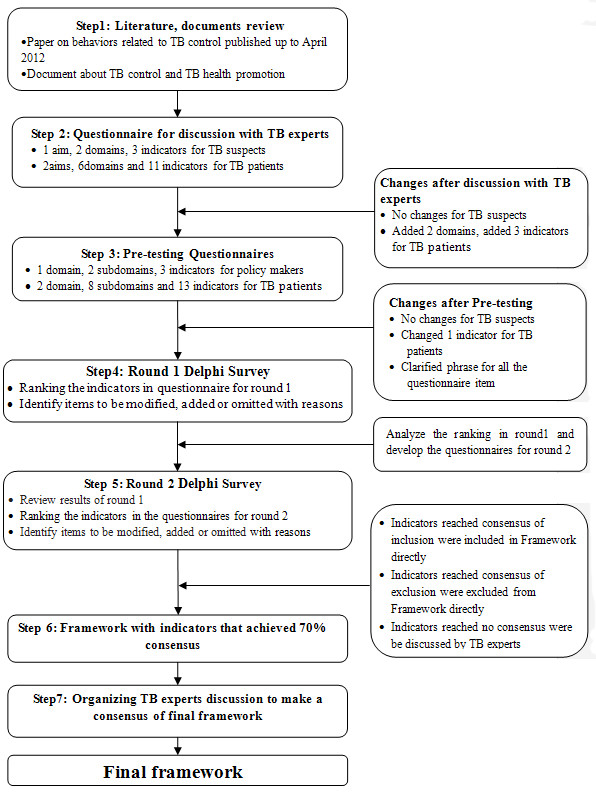
**Flowchart of the Delphi survey questionnaire design and Delphi process.** This figure describes the process in the development of main Delphi survey questionnaire (Steps 1–3) and the procedure used to implement the Delphi process (Steps 4–7).

The process of development of the main Delphi survey questionnaire is presented in Figure 1 (steps 1-3). First, we developed a detailed framework that was created based on an in-depth review of TB experts, information from previous studies [11,47-54], and review of relevant literature on TB control and TB health promotion [17,28,55]. Second, TB health professionals working in TB dispensaries were asked to comment on the draft instrument, and to add as appropriate, other indicators and operational definitions. This resulted in two draft questionnaires (domains, subdomains, and indicators) on TB health promotion, one for TB suspects and one for TB patients. Third, the questionnaires were pre-tested on a convenient sample of 3 TB health workers who reviewed appropriateness and clarity of the questionnaire items. Fourth, as a result of pre-testing, proper phrasing was developed; respondents’ interpretations were evaluated, and Delphi questionnaires were finalized and applied as the survey instruments used to obtain responses from experts. The Delphi survey questionnaire that addressed health promotion indicators for TB suspects included 1 domain, 2 subdomains, and 3 indicators. The survey instrument for TB patients included 2 domains, 8 subdomains, and 14 indicators (Additional file 1: Table S1). Both instruments included operational definitions of all the potentially relevant indicators. Experts were asked to assess the importance and feasibility of each indicator on a 5-point Likert Scale in the following order and score: extremely unimportant/infeasible (1), unimportant/infeasible (3), somewhat important/feasible (5), important/feasible (7), and extremely important/feasible (9). Finally, the experts were given the options to delete, add, and modify indicators but were required to articulate the reasons behind their choices.

Information on experts’ authority included levels of familiarity with the indicators (extremely familiar, very familiar, somewhat familiar, somewhat unfamiliar, very unfamiliar, extremely unfamiliar) and judgment criteria on these indicators (theory analysis, practical experience, literature review, intuition) [56]. General information about the experts included age, position, professional title, education, work area, and years of experience in TB control.

### Setting the consensus level

The Delphi method is based on panelists’ achieving consensus. However, expert opinions can differ, and it is difficult to gain 100% agreement on all issues. Therefore, a key question in Delphi studies is the percentage of agreement the researcher would accept as representing consensus [57]. However, no standard method or appropriate guidelines are available for determining consensus levels [57-60]. For this study and in line with other related studies [61-69], we set the consensus level at 70% as follows:

1) Consensus of inclusion: >70% of participants scored the item ≥7;

2) Consensus of exclusion: > 70% subjects scored the item ≤5;

3) No consensus: item failed to meet either of the above criteria.

### Procedures for the Delphi survey

Figure 1 shows the flow of the Delphi process (Step 4–7). In the first round, participants were asked to rank the importance and feasibility of the indicators to evaluate behavioral outcomes of individual level TB health promotion. The responses in the first-round survey were analyzed, using descriptive statistics, and the results were sent back to the experts for review and ratification. Items that achieved consensus of exclusion (i.e., > 70% of the experts scored the item ≤5) in the first round survey were excluded from the questionnaire for second round survey. Items for which were recommended for modification by the experts were revised and added to the second round survey; new items suggested by the experts were also added to the second round survey. In the second survey, participants were asked to re-rank the consensus results from the first round. Second Delphi responses that reached 70% consensus were determined as appropriate items (domains, subdomains and indicators) for assessing behavioral outcomes of individual level TB health promotion. The final framework was presented to experts for discussion and final consensus, leading to the final framework.

### Statistical analysis

Descriptive data analyses for the first and second round Delphi surveys were undertaken, using the Statistical Package for the Social Sciences (SPSS) version 18.0. The authority coefficient (C_r_) was used to assess the degree of the experts’ authority in relation to their technical ability to evaluate the indicators as determined by two factors, the judgment criteria for the indicators (C_a_) (Additional file 1: Table S2) and the experts’ familiarity with the indicators (C_s_) (Additional file 1: Table S3) [56]. C_r_ is defined as follows:

$$C_{r}=\frac{C_{a}+C_{s}}{2}$$

Median and mode were used to describe the central tendency of expert responses.

Coefficient of variation (CV) was used to describe the dispersals of expert responses. CV is the ratio of the standard deviation of the responses of the experts on a specific item to its corresponding mean (average). Therefore, the responses of the experts for each survey item in each round of the Delphi survey yielded one CV [70].

## Results

### Characteristics of the experts

**Figure 2:**
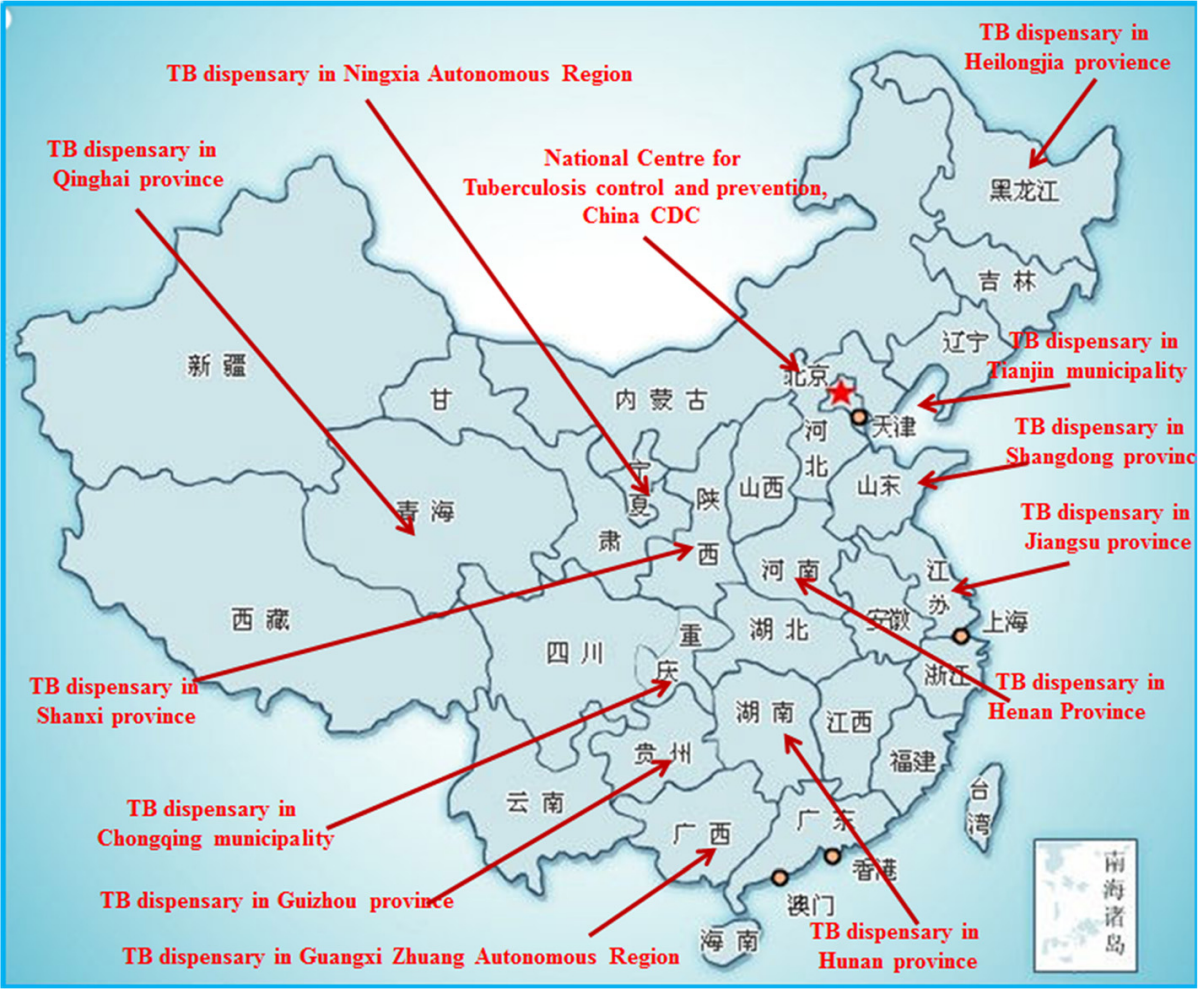
**Experts in Delphi expert panel.** This figure presents the geographic distribution of experts who participated in the Delphi expert panel. The sixteen experts were from thirteen provinces/regions representing North, South, East, and West, China.

During the first round Delphi survey, questionnaires were sent to seventeen experts, sixteen of whom responded. The sixteen experts who participated in the survey were from thirteen provinces/regions representing North, South, West, and East China (Figure 2). All sixteen experts who responded to the first round survey also completed the second round survey. Descriptive information about the experts is presented in Table 1. All the experts had 8 or more years of experience (ranging from 8 to 36; mean = 11.9, SD = 5.1) as full-time professionals in TB control facilities. A majority (n = 12) had 11 to 30 years of experience.

**Table 1:** Expert characteristics

**Characteristics**	**Number**	**Percentage**
**Age**		
<40	1	6.3
40-45	3	18.8
45-50	9	56.3
>50	3	18.8
**Years of experience in TB control**		
≤10	2	12.5
11-20	5	31.3
21-30	7	43.8
>30	2	12.5
**Types of expertise**		
Policy makers	3	18.8
Managers in TB dispensaries	9	56.3
Staff in TB dispensaries	5	31.3
**Work unit**		
National level	3	18.8
Province level	13	81.3
**Educational level**		
College	2	12.5
University	10	62.5
Graduate	4	25.0
**Professional title**		
Middle	2	12.5
Associate senior	5	31.3
Senior	9	56.3
**Response rates to questionnaires**		
Round 1	16	94.1
Round 2	16	100

### Experts’ authority levels (Cr)

The expert authority coefficient ranged from 0.91 to 0.92, and the average Cr was 0.92, which indicated that all 16 experts had high degree of authority in the area of TB control and prevention, which clearly qualifies them to evaluate indicators (Table 2).

**Table 2:** Expert levels of authority

**Round**	**Judgment criterion (C**_**a**_**)**	**Familiarity (C**_**s**_**)**	**Authority (C**_**r**_**)**
First round	0.92	0.91	0.91
Second round	0.93	0.91	0.92
Average	0.92	0.91	0.92

### Results of round-1 survey

Results of round 1 Delphi survey of the experts are shown in Table 3. Based on rankings for importance and feasibility for the framework for TB-suspect by experts, median scores ranged from 7 to 9, and CVs were less than 0.3 (Table 3). Those results indicated that expert rankings had good central tendencies for all items in terms of indicator’s importance and feasibility.

**Table 3:** Results of round-1 survey

**Items**	**Importance**				**Feasibility**			
	**Median**	**Mode**	**CV**	**Consensus (% score of >7)**	**Median**	**Mode**	**CV**	**Consensus (% score of >7)**
**TB suspects**								
***Domain***								
Health-care seeking behavior	9	9	0.1	93.8	7	7	0.2	81.3
***Subdomain***								
Care-seeking behavior at onset of TB symptoms	9	9	0.0	100	7	7	0.2	81.3
Care-seeking pathways	9	9	0.1	100	7	7	0.2	75.1
***Indicators***								
Length of patient delay and percentage of patients with longer patient delay	9	9	0.1	100	7	7	0.2	62.6
Average number of health care provider encounters before diagnosis and percentage of patients who encountered ≥2 non-TB health providers	7	7	0.2	75.1	7	7	0.2	81.3
Percentage of patients with first non-TB health contact following onset of TB symptoms	8	9	0.2	93.8	9	9	0.2	81.3
**TB patients**								
***Domain***								
Adherence to treatment	9	9	0.0	100	9	9	0.16	93.8
Isolation behaviors during period of infection	9	9	0.2	83.8	5	7	0.5	43.8
***Subdomain***								
Adhere to medication	9	9	0.0	100	8	9	0.2	75
Follow-up sputum microscopy	9	9	0.1	100	9	9	0.1	93.8
Changing unhealthy lifestyle	7	9	0.2	68.8	5	5	0.3	37.5
Disposal of sputum	9	9	0.2	83.4	7	9	0.3	68.8
Wearing respirator in public during infective phase of disease	7	9	0.2	66.7	5	5	0.5	40
Behaviors related to using separate utensils (spoons, plates, forks and chopsticks)	5	5	0.6	33	8	9	0.4	62.5
Isolation room	7	7	0.2	66.6	7	7	0.4	62.6
Room ventilation	7	9	0.1	100	7	7	0.3	66.7
***Indicators***								
Percentage of patients who adhered to their medication	9	9	0.0	100	7	7	0.2	80
Percentage of patients who missed dose	8	9	0.1	100	7	7	0.2	87.5
Percentage of patients with interrupted treatment	9	9	0.1	100	7	9	0.2	65
Percentage of patients with default treatment	9	9	0.3	93.8	7	9	0.4	68.8
Percentage of patients who kept follow-up sputum microscopy appointment	9	9	0.1	100	9	9	0.2	93.8
Percentage of patients who abstained from smoking	7	9	0.3	68.8	5	5	0.4	43.8
Percentage of patients who abstained from alcohol drinking	7	9	0.3	66.6	5	5	0.4	33.4
Percentage of patients who consistently abstained from spitting	9	9	0.1	100	7	9	0.4	62.5
Percentage of patients with safe method of sputum disposal	9	9	0.1	100	7	9	0.4	62.6
Percentage of patients who covered face/nose when sneezing/cough/speaking loudly	9	9	0.1	100	7	9	0.4	56.3
Percentage of patients who often wore respirator in public	7	9	0.2	75	5	5	0.4	50
Percentage of patients who used dishes and chopsticks separately	5	5	0.5	43.8	9	9	0.2	75.1
Percentage of patients with isolation room	7	7	0.2	75	7	9	0.4	56.3
Percentage of patients who often ventilated room	9	9	0.1	100	9	9	0.2	87.6

Note: CV refers to Coefficient of variation.

For the TB patients, medians and modes for the importance were 7 or 9 and CVs for the importance were lest 0.3 except for two items (“Behaviors related to deal with dishes”, and “Percentage of patients who used dishes and chopsticks separately”). However, the median for feasibility rankings of 6 items and mode for feasibility rankings of 5 items were 5; CVs for feasibility rankings of 12 items were more than 0.3 (Table 3). These results indicated that expert rankings had good central tendencies for most items in terms of importance, but not for the feasibility.

### Indicator screening after first-round survey

Based on criteria for the consensus as earlier stated, indicators with a consensus score of >7 by >70% of the experts were considered as appropriate indicators and indicators with a consensus score of ≤5 by >70% of experts were excluded. Table 3 shows that for TB suspects, inclusion consensus was reached after round-1 survey on: one domain (“Health-care seeking behavior”), two sub-domains (“Care seeking behavior at onset of TB symptoms” and “Care-seeking pathways”) and two indicators (“Average number of health care provider encounters before diagnosis and percentage of patients who encountered ≥2 non-TB health providers” and “Percentage of patients with first non-TB health contact following onset of TB symptoms”).

For TB patients, consensus was reached on: one domain (“Adherence to treatment”), two sub-domains (“Adherence to their medication” and “Follow-up sputum microscopy”), and four indicators (“Percentage of patients who adhere to their medication”, “Percentage of patients who missed dose”, “Percentage of patients who kept follow-up sputum microscopy” and “Percentage of patients who often ventilated room”).

Regarding the indicators for TB suspects, experts did not suggest deleting any item; two indicators (“Period of patient delay and percentage of patients with longer patient delay” and “Average number of health care provider encounters before diagnosis and percentage of patients who encountered ≥2 health providers”) were suggested to be separated into two indicators for each; five new items (1 domain, 1 subdomain and 3 indicators) were suggested to be added to the questionnaire (Additional file 1: Table S4). Finally, 2 domains, 3 subdomains, and 8 indicators were included in the questionnaire for the second-round survey (Table 3).

As for indicators for TB patients, experts in the first-round survey suggested the deletion of: 4 subdomains (“Change unhealthy lifestyle”, “Wearing respirator in intensive phrase in public”, “Behaviors related to deal with dishes”, and “Isolation room”), and 2 indicators (“Percentage of patients who used dishes and chopsticks separately” and “Percentage of patients who had separate living room from others in household”). One item in the domain was modified (“Isolation behaviors” was changed into “Transmission prevention”). They also suggested the inclusion of eight new items to the questionnaire, including 1 domain, 4 sub-domains, and 3 indicators (Additional file 1: Table S4). Finally, 3 domains, 8 sub-domains, and 15 indicators were included in the questionnaire for the second-round survey (Table 3).

### Results of second survey

According to the rankings in the round-2 survey, items in the questionnaire for both TB suspects and TB patients were very important and feasible (median or mode for importance and feasibility for all indicators ranged from 7 to 9, CVs for all indicators were less than 0.3) (Table 4). These results indicated that expert rankings in the second round survey had good central tendency for most of the items in terms of importance and feasibility for both TB suspects and TB patients.

**Table 4:** Results of round 2 survey

**Item**	**Importance**				**Feasibility**			
	**Median**	**Mode**	**CV**	**Consensus (% score of >7)**	**Median**	**Mode**	**CV**	**Consensus (% score of >7)**
**TB suspect**								
***Domain***								
Healthcare seeking behavior	9	9	0.1	100	7	7	0.1	100
Transmission prevention	9	9	0.1	100	7	7	0.2	68.8
***Subdomain***								
Seeking care after onset of TB symptom	9	9	0.0	100	9	9	0.1	100
Pathways of seeking care	9	9	0.1	100	7	7	0.1	93.8
Interpersonal contact etiquette	9	9	0.2	93.7	7	7	0.4	56.3
***Indicator***								
Length of patient delay and	9	9	0.1	100	7	9	0.2	87.6
Percentage of patients with longer patient delay	9	9	0.1	100	7	9	0.2	87.6
Number of health care provider encounters before diagnosis, and	7	7	0.2	87.6	8	9	0.2	81.3
Percentage of patients who encountered ≥2 non-TB health providers	7	7	0.2	87.6	8	9	0.2	81.3
Percentage of patients with first non-TB health contact following onset of TB symptoms	9	9	0.1	100	9	9	0.2	87.6
Percentage of patients who wore respirator in public during in infective phrase of disease	7	9	0.2	81.3	7	9	0.2	75.1
Percentage of patients who consistently abstained from spitting	9	9	0.2	93.7	7	7	0.3	81.3
Percentage of patients who covered face when sneezing/cough/speaking loudly	9	9	0.1	100	7	7	0.3	75
**TB patient**								
***Domain***								
Adherence to treatment	9	9	0.0	100	9	9	0.1	100
Healthy lifestyle	9	9	0.2	100	7	7	0.2	68.8
Transmission prevention	9	9	0.1	93.7	7	7	0.2	75
***Subdomain***								
Adhere to medication	7	9	0.1	100	9	9	0.1	100
Follow-up sputum microscopy	9	9	0.1	100	9	9	0.1	100
Abstinence from smoking	7	7	0.2	78.3	7	7	0.2	81.3
Abstinence from alcohol drinking	7	9	0.2	87.5	7	7	0.2	75.1
Nutrition improvement	7	9	0.2	93.3	7	7	0.3	73.4
Disposal of sputum	9	9	0.1	100	9	9	0.2	81.3
Interpersonal contact etiquette	9	9	0.2	93.7	7	7	0.3	68.8
Room ventilation	9	9	0.1	100	9	9	0.2	98.7
***Indicator***								
Percentage of patients who adhered to their medication	9	9	0.0	100	9	9	0.1	93.7
Percentage of patients who missed dose of drugs	9	9	0.1	100	9	9	0.2	93.7
Percentage of patients with interrupted treatment	9	9	0.1	100	9	9	0.2	100
Percentage of patients with default treatment	9	9	0.2	93.3	9	9	0.1	100
Percentage of patients who kept follow-up sputum microscopy	9	9	0.0	100	9	9	0.1	100
Percentage of patients who abstained from smoking	7	7	0.2	78.3	7	7	0.2	81.3
Percentage of patients who abstained from alcohol drinking	7	9	0.2	87.5	7	7	0.2	75.1
Percentage of patients who improved nutrition	7	9	0.2	93.3	7	7	0.3	73.4
Percentage of patients who consistently abstained from spitting	9	9	0.1	100	7	9	0.2	81.3
Percentage of patients with safe method of sputum disposal	9	9	0.2	93.3	9	9	0.2	86.6
Percentage of patients who covered face when sneezing/cough/speaking loudly	9	9	0.1	100	7	9	0.2	75.1
Percentage of patients who wore respirator in public during in infective phrase of disease	9	9	0.2	87.6	7	7	0.3	62.5
Percentage of patients who reduced frequency of presence in public	8	9	0.2	68.8	7	7	0.2	62.6
Percentage of patients who informed contact of TB status	8	9	0.1	100	7	9	0.2	100
Percentage of patients who ventilated their room	9	9	0	100	9	9	0.1	100

Note: CV refers to Coefficient of variation.

### Framework of indicators for behavioral outcome assessment of TB health promotion for TB suspects and patients

The following results are based on our criteria for inclusion and exclusion consensus. Regarding indicators for TB suspects, consensus was achieved for importance and feasibility except the feasibility of one domain (“Transmission prevention”, scored ≥7 by 68.8%) and one subdomain (“Interpersonal contact etiquette”, scored ≥7 by 56.3%) (Table 4). For TB patients, only one indicator (“Percentage of patients who reduced frequency of presence in public”) was scored ≥7 by <70% participants for both importance and feasibility (68.8% and 62.6%). Consensus was achieved on importance of the rest items and on feasibility of most items except for one domain (“Healthy lifestyle”, scored ≥7 by 68.8%), one subdomain (“Interpersonal contact etiquette”, scored ≥7 56.3%), and one indicator (“Percentage of patients who wear respirators in public”, scored ≥7 62.5%) (Table 4).

We organized a round-table discussion for TB control experts to decide on final items for the framework. Following this discussion, 2 domains, 3 subdomains, and 8 indicators emerged for TB suspects. There 3 domains, 8 subdomains, and 14 indicators emerged for TB patients (Table 5). In addition, we provided definitions related to the indicators in Additional file 1: Table S5.

**Table 5:** Framework of indicators

**Domain**	**Subdomains**	**Indicators**	**Measures**	**Data source**
***TB suspects***				
Healthcare seeking behavior	Seeking care after onset of TB symptoms	Length of patient delay	Mean, median and range of patient delay	TB suspect survey and clinic record review to learn the time of TB symptoms onset and the time for seeking care for first time
		Percentage of patients with longer patient delay	Percentage of patients with longer delay	TB suspect survey and clinic record
	Pathways of seeking care	Average number of health care provider encounters before diagnosis	Average number of health care provider encounters before diagnosis	TB suspect survey and clinic record
		Percentage of patients who encountered ≥2 non-TB health providers	Percentage of patients who encountered ≥2 non-TB health providers	TB suspect survey and clinic record
		Percentage of patients with first non-TB health contact following onset of TB symptoms	Percentage of TB suspects who had first provider contact in non-TB health facility after onset of TB symptoms	TB suspect survey and clinic record
Transmission prevention	Interpersonal contact etiquette	Percentage of patients who wore respirator in Public	Percentage of patients who wore respirator in Public after onset of TB symptoms	TB suspect survey
		Percentage of patients who consistently abstained from spitting	Percentage of patients who consistently abstained from spitting after onset of TB symptoms	TB suspect survey
		Percentage of patients who consistently covered face when sneezing/cough/speaking loudly	Percentage of patients who consistently covered face when sneezing/cough/speaking loudly	TB suspect survey
***TB patients***				
Adherence to treatment	Adherence to medication	Percentage of patients who adhered to medication	Percentage of patients who adhered to their medication	TB patient survey and clinic record review
		Percentage of patients with interrupted treatment	Percentage of patients who experienced interrupted treatment.	TB patient survey and clinic record review
		Percentage of patients with default treatment	Percentage of patients who experienced default treatment.	TB patient survey and clinic record review
	Follow-up sputum microscopy	Percentage of patients who kept follow-up sputum microscopy	Percentage of patients who kept follow-up sputum microscopy at the end of 2, 5 and 6 month of treatment	TB patient survey or and clinic record review
Healthy lifestyle	Abstinence from smoking	Percentage of patients who abstained from smoking	Percentage of patients who abstained from smoking following TB health promotion.	TB patient survey
	Abstinence from alcohol drinking	Percentage of patients who abstained from alcohol drinking	Percentage of patients who abstained from drinking alcohol following TB health promotion.	TB patient survey
	Nutrition improvement	Percentage of patients with improved nutritional status	Percentage of patients with improved nutritional status (e.g., assessed through weight or other anthropometric measures) following TB health promotion.	TB patient survey
Transmission prevention	Disposal of sputum	Percentage of patients who consistently abstained from spitting	Percentage of patients who consistently abstained from spitting sputum after diagnosis with TB	TB patient survey
		Percentage of patients with safe method of sputum disposal	Percentage of patients with safe method of sputum disposal during the infective phrase of disease	TB patient survey
	Interpersonal contact etiquette	Percentage of patients who covered face when sneezing/cough/speaking loudly	Percentage of patients who consistently covered face among ten times of sneezing/cough/speaking loudly	TB patient survey
		Percentage of patients who wore respirator in public during in infective phrase of disease	Percentage of patients who consistently wore respirator in public after diagnosis of TB	TB patient survey
		Percentage of patients who reduced frequency of presence in public	Percentage of patients who reduce times spent in public places	TB patient survey
		Percentage of patients who informed contact of TB status	Percentage of patients who disclosed their TB infection to their contacts and informed them to screen for TB	TB patient survey and clinic record review about contact screening
	Room ventilation	Percentage of patients who ventilated their room	Percentage of patients who often ventilated their living room	TB patient survey

## Discussion

Health promotion has the capacity to address most of the TB prevention and care challenges at the individual, societal, and health systems levels. However, to demonstrate the utility of health promotion in efforts to reduce the global burden of TB, appropriate indicators which can facilitate evaluation of its outcomes are urgently needed. Although there are compendiums of indicators for assessing interventions to address such other major infectious diseases of global significance as HIV/AIDS [32,33], there are currently no validated indicators to guide implementation and evaluation of behavioral interventions to reduce TB. In an attempt to address this gap in knowledge and practice, we embarked on the development of such a compendium of behavioral indicators for TB health promotion interventions, using the Delphi method, which has been widely applied in diverse areas of population health [67,71].

Participants in the Delphi surveys and discussions were TB control experts with extensive field experience. The authority coefficient was 0.91 in the first-round Delphi survey and 0.92 in the second-round survey, which indicated a high degree of authority in the field of TB control of the experts in the Delphi surveys, and which qualified them for participation in the survey.

The Delphi process benefitted from the use of a survey instrument that was developed following a systematic review of the global TB control literature [11,47-60], review of government TB control program documents [28], local and international expert consultations. According to documents review, one of the behavioral goals of TB health promotion is to encourage TB suspects to seek healthcare in a timely manner, and to adhere to treatment and management of their disease [15,28,55]. Consequently, the conceptual framework proposed in this study included 1 domain (“Healthcare seeking behavior”), 2 sub-domains (“Seeking care after onset of TB symptoms” and “Pathways of seeking care”) and 5 indicators (including among other, “Length of patient delay”) to evaluate the healthcare seeking behaviors for TB suspects. To evaluate health promotion activities that target behaviors related to adherence to treatment for TB patients, the proposed framework included 1 domain (“Adherence to treatment”), 2 sub-domains (“Adherence to medication” and “Follow-up sputum microscopy”) and 4 indicators (including among other, “Percentage of patients who adhered to their medication”).

TB is a communicable disease that spreads through the air. If untreated, each patient with active TB can on average, infect 10 to 15 people every year [72]. It is therefore, important for TB suspects and patients to adopt behaviors that discourage transmission of the infection to others through TB health promotion [15,55]. Thus, in order to evaluate outcome of TB health promotion in TB prevention, this study proposed a framework that includes for TB suspects, 1 domain (“Transmission prevention”), 1 sub-domain (“Interpersonal contact etiquette”) and 3 indicators (including among others, “Percentage of patients who wore respirator in Public”). The framework for TB patients includes 1 domain (“Transmission prevention”), 2 sub-domains (“Interpersonal contact etiquette” and “Room ventilation”) and 5 indicators (including among others, “Percentage of patients who consistently covered their mouth/nose when sneezing/cough/speaking loudly”).

Although TB health promotion primarily aims to encourage patients to adhere to their treatment, WHO documents also emphasized that efforts should be made to cover a wider range of health related behaviors that help to prevent and cure TB, such as improved nutrition, avoidance of smoking, abstinence from alcohol use, as well as behaviors that prevent TB [15,55]. Evidences from the literature shows that smoking, use of alcohol and poor nutrition are associated with poor TB treatment outcome [53,54]. Therefore, for TB patients, the proposed framework includes 1 domain (Healthy lifestyle), 3 sub-domains (“Abstaining from smoking”, “Abstaining from alcohol drinking” and “Nutrition improvement”) and 3 indicators (“Percentage of patients who abstained from smoking”, “Percentage of patients who abstained from alcohol drinking” and “Percentage of patients who improved nutrition”).

### Strengthens and limitations

This study fills an important void in efforts to control TB globally by suggesting benchmarks that TB control programs around the world, which could adopt in their assessment of outcomes of their efforts. In the face of scarcity of resources, and considering that every resource spent on TB health promotion has opportunity costs in other sectors of any country’s economy, it is important to determine whether TB health promotion efforts are making an impact. This is critical in determining if resources are being spent judiciously or whether the resource should be spent on alternative strategies of proven effectiveness.

The Delphi process adopted in this study employed strict quality control measures. Local and international TB control experts were consulted in the design and implementation of the Delphi surveys. The expert panel consisted of professionals from thirteen provinces of China and demonstrated a high authority coefficient. Most experts had leading positions within their institutions in addition to extensive experience in TB control. Due to the multiple feedback processes inherent in the Delphi process, the potential low response rates, and striving to maintain robust feedback can be a challenge [59]. However, the return rates for this study were high at 94.1% and 100% for round 1 and 2 surveys, respectively.

Although the experts achieved high consensus on the domains, sub-domains and indicators, it is important to note that the extent to which participants agree with each other does not necessarily mean that the “correct” answer has been found [56]. Thus, there is the danger of over reliance on the final results without acknowledging the influence of bias and other factors on validity and reliability [56]. Further validation of the identified indicators by other national and global TB control programs is therefore warranted. It is also important to note that as a qualitative research method, Delphi studies do not, and are not intended to, produce statistically significant results. Rather, the results represent a synthesis of the opinions of the particular group involved in the process [73]. Thus, findings from this Delphi survey represent an important starting point for debate and consensus on global indicators for assessing the effectiveness of TB behavioral health promotion programs. The involvement of more experts and TB control programs from other parts of the globe will increase validity and reliability of the results [58]. To enhance generalizability, a number of strategies can be used: focus groups can be integrated or comparisons can be made with sub-domain-validated data [56]. A quasi-experimental design can be used as a follow-up method to test the results of the Delphi process [61], and a “consensus conference” can be organized to discuss the validity of the Delphi results. Finally, as noted in Table 5, much of the evaluation data for the proposed framework of indicators would rely on reports from patients and suspects. Since self-reports are known to be subject to social desirability, it is important that evaluation strategies for TB health promotion include good quality control and mixed-method approaches that seek to reduce bias and increase data quality.

## Conclusion and implications

This investigation proposes a framework of primary behavioral indicators for evaluation of TB health promotion programs. The results provide a basis for further research. Before any performance indicator can be adopted, it needs to be clearly defined and tested for reliability, validity, and responsiveness (the ability to detect a significant change in performance). Therefore, further studies are needed to validate the indicators in diverse settings. In spite of the need for further study, the proposed framework of TB behavioral health promotion indicators can be used to more comprehensively monitor, evaluate, and improve the quality of TB health promotion programs by health practitioners and policy makers.

## Abbreviations

MDR-TB: Multi-drug resistant TB; HIV/AIDS: Human immunodeficiency virus infection/acquired immunodeficiency syndrome; SPSS: Statistical package for the social sciences; CV: Coefficient of variation; SD: Standard deviation; WHO: World Health Organization.

## Competing interests

The authors declare that they have no competing interests.

## Authors’ contributions

YL designed the study. DH and YL designed the instrument for data collection and organized expert discussion; YL and YZ organized and analyzed data; DH, and JC contacted the experts for Delphi survey. YL and JE drafted the manuscript. JE revised and edited the manuscript. All authors interpreted the results, commented on the report, and approved the final version.

## Pre-publication history

The pre-publication history for this paper can be accessed here:

http://www.biomedcentral.com/1471-2334/14/268/prepub

## Supplementary Material

Additional file 1: Table S1 Items in the questionnaires of round 1 Delphi survey. **Table S2.** Quantification of judgment criterion. **Table S3.** Quantification of the level of familiarity. **Table S4.** Indicators changes after first round survey. **Table S5.** Definitions of indicators. Click here for file
