# Supplementary material for: Framework of behavioral indicators for outcome evaluation of TB health promotion: a Delphi study of TB suspects and Tb patients
Source: BMC Infect Dis. 2014 May 16;14:268. doi: 10.1186/1471-2334-14-268 (PMC4030006; doi:10.1186/1471-2334-14-268)
Supplement: Additional file 1: Table S1 — Items in the questionnaires of round 1 Delphi survey. Table S2. Quantification of judgment criterion. Table S3. Quantification of the level of familiarity. Table S4. Indicators changes after first round survey. Table S5. Definitions of indicators. [file 1471-2334-14-268-S1.docx]

**Additional file 1**

**Table S1 Items in the questionnaires of round 1 Delphi survey**

| **Domain** | **Subdomains** | **Indicators** | **Measures** |
| --- | --- | --- | --- |
| ***TB suspects*** | | | |
| Healthcare seeking behavior | Care seeking behavior at onset of TB symptoms | Period of patient delay and percentage of patients with longer patient delay | Patient delay refers to the time interval between the onset of TB symptoms and the patient’s first presentation to a health facility (excluding buying medicine from pharmacy). Longer patient delay refers to the patient delay ≥ the Median value of patient delay |
|  | Care seeking pathways | Average number of health care provider encounters before diagnosis and percentage of patients who encountered ≥2 health providers | Average number of non-TB health care provider encounters before diagnosis (excluding buying medicine from pharmacy and seeking care in TB control health facilities) and percentage of patients who encountered ≥2 non-TB health providers |
|  |  | Percentage of patients with first provider contact in non-TB health facility following onset of TB symptoms | Percentage of TB suspects who had first provider contact in non-TB health facility after onset of TB symptoms (excluding buying medicine from pharmacy) |
| ***TB patients*** | | | |
| Adherence to treatment | Adhere to their medication | Percentage of patients who adhere to their medication | Percentage of patients who regularly take anti-TB drugs prescribed by doctors |
|  |  | Percentage of patients with missed dose | Percentage of patients who missed doses of anti-TB drugs. Missed dose is defined as TB patients forget take anti-TB drugs for 1-6 times in one month, but less than 6 times according to the recommended course of treatment. |
|  |  | Percentage of patients with interrupted treatment | Percentage of patients who experienced interrupted treatment. Interrupted treatment is defined as Patients who discontinued medication for 2-8 consecutive weeks but eventually restarted treatment were considered to have interrupted treatment**.** |
|  |  | Percentage of patients with default | Percentage of patients who experience default treatment. Default is defined as patient who interrupted treatment for more than 2 months consecutively. |
|  | Keeping follow-up appointments with care providers | Percentage of patients who kept follow-up appointments with care providers | Percentage of patients who take regularly follow-up spu­tum check. Follow-up spu­tum check refers patients should come to take spu­tum check at the end of 2, 5 and 6 month of treatment. |
|  | Change unhealthy lifestyle | Percentage of patients who abstained from smoking | Percentage of patients who topped smoking among the smoking TB patients after diagnosis of TB |
|  |  | Percentage of patients who abstained from alcohol drinking | Percentage of patients who topped drinking among the alcohol drinking TB patients after diagnosis of TB |
| Isolation behaviors during period of infection | Disposal of sputum | Percentage of patients who consistently abstained from spitting | Percentage of patients who had no habit of no spitting sputum after diagnosis with TB |
|  |  | Percentage of patients with safe method of sputum disposal | Percentage of patients with safe method of sputum disposal who managed sputum they coughed according to the manual of TB control during in infective phrase of disease |
|  |  | Percentage of patients who consistently covered mouth/nose when sneezing /cough /speaking loudly | Percentage of patients who covered mouth/nose among ten times of sneezing/cough/speaking loudly and percentage of patients who cover mouth/nose among ten times of sneezing/cough/speaking loudly |
|  | Wearing respirator intensive phrase in public | Wearing respirator in public during in infective phrase of disease | Percentage of patients who often wearied respirator in public after diagnosis of TB |
|  | Behaviors related to using separate utensils (spoons, plates, forks and chopsticks) | Percentage of patients who used dishes and chopsticks separately | Percentage of patients who used dishes and chopsticks separately after diagnosis of TB |
|  | Isolation room | Percentage of patients with isolation room | Percentage of patients who had single patient room with negative pressure ventilation where an infectious TB patient can be isolated from other patients. had separate living room after diagnosis of TB |
|  | Room ventilation | Percentage of patients who often ventilated room | Percentage of patients who often ventilated room after diagnosis of TB |

**Table S2 Quantification of judgment criterion**

| Judgment criterion | Value | | |
| --- | --- | --- | --- |
|  | Large | Middle | Small |
| Theory analysis | 0.3 | 0.2 | 0.1 |
| Practice experience | 0.5 | 0.4 | 0.3 |
| Literature review | 0.1 | 0.1 | 0.1 |
| Intuition | 0.1 | 0.1 | 0.1 |

**Table S3 Quantification of the level of familiarity**

| Level of familiarity | Value |
| --- | --- |
| Extremely familiarity | 1.0 |
| very familiarity | 0.8 |
| somewhat familiarity | 0.6 |
| somewhat unfamiliarity | 0.4 |
| very unfamiliarity | 0.2 |
| extremely unfamiliarity | 0 |

**Appedix4 Indicators changes after first round survey**

| Changes | Domains | Subdomains | Indicators |
| --- | --- | --- | --- |
| ***TB suspect*** | | | |
| Added | Transmission prevent | Interpersonal contact etiquette | Percentage of patients who consistently wearied respirator in public, Percentage of patients who consistently abstained from spitting,  Percentage of patients who covered face when sneezing /cough/speaking loudly |
| Modified |  |  | “Period of patient delay and percentage of patients with longer patient delay” was changed into two indicators (“Period of patient delay” and “percentage of patients with longer patient delay”); “Average number of health care provider encounters before diagnosis and percentage of patients who encountered ≥2 health providers” was changed into two indicators (“Average number of health care provider encounters before diagnosis” and percentage of patients who encountered ≥2 health providers before diagnosis ) |
| Deleted |  |  |  |
| ***TB patients*** | | | |
| Added | Healthy lifestyle | Abstinence from smoking from smoking,  Abstinence from alcohol drinking,  Nutrition improvement, Interpersonal contact etiquette | Percentage of patients who reduced frequency of presenting the public,  Percentage of patients who informed contact of TB status,  Percentage of patients who ventilated of room |
| Modified | Isolation behaviors was changed into transmission prevention |  |  |
| Deleted |  | Changing unhealthy lifestyle,  Wearing respirator in intensive phrase in public, Behaviors related to using separate utensils (spoons, plates, forks and chopsticks), Percentage of patients with isolation room | Percentage of patients who used dishes and chopsticks separately,  Percentage of patients who had separate living room from others in household |

**Table S5** **Definitions of indicators**

| Box of definitions |
| --- |
| **Patient delay** refers to the time interval between the onset of TB symptoms and the patient’s first presentation to a health facility (excluding buying medicine from pharmacy) *[Jurcev-Savicevic A, Kardum G.Health-care seeking behaviour for tuberculosis symptoms in Croatia. Eur J Public Health. 2012 Aug;22(4):573-7.]* |
| **Longer patient delay** refers to patient delay ≥ the Median value of patient delay*[*[*Jurcev-Savicevic A*](http://www.ncbi.nlm.nih.gov/pubmed?term=Jurcev-Savicevic%20A%5BAuthor%5D&cauthor=true&cauthor_uid=21920849)*,* [*Kardum G*](http://www.ncbi.nlm.nih.gov/pubmed?term=Kardum%20G%5BAuthor%5D&cauthor=true&cauthor_uid=21920849)*.Health-care seeking behaviour for tuberculosis symptoms in Croatia.* [*Eur J Public Health.*](http://www.ncbi.nlm.nih.gov/pubmed/?term=Health-care+seeking+behaviour+for+tuberculosis+symptoms+in+Croatia) *2012 Aug;22(4):573-7. ]* |
| **Health providers** is non-TB health providers who work in health centers, hospitals & clinics owned by the government or the private sectors rather than health facility belong to National TB program, excluding buying medicine from pharmacy) *[Yimer S, Bjune G, Alene G. Diagnostic and treatment delay among pulmonary tuberculosis patients in Ethiopia: a cross sectional study. BMC Infect Dis. 2005 Dec 12;5:112.]* |
| **Non-TB health providers** refers to health providers in health centers, hospitals & clinics owned by the government or the private sectors rather than health facility belong to National TB program, excluding buying medicine from pharmacy) *[Yimer S, Bjune G, Alene G. Diagnostic and treatment delay among pulmonary tuberculosis patients in Ethiopia: a cross sectional study. BMC Infect Dis. 2005 Dec 12;5:112.]* |
| **Non-TB health facility** refers to health centers, hospitals & clinics owned by the government or the private sectors rather than health facility belong to National TB program, excluding buying medicine from pharmacy (excluding buying medicine from pharmacy) *[Yimer S, Bjune G, Alene G. Diagnostic and treatment delay among pulmonary tuberculosis patients in Ethiopia: a cross sectional study. BMC Infect Dis. 2005 Dec 12;5:112.]* |
| **Respirator** refers to N95 respirator or equivalents *[Source: CDC. Guidelines for preventing the transmission of Mycobacterium tuberculosis in health-care facilities, 2005. MMWR 2005;54(No. RR-17):38–40.]* |
| Abstinence from spitting |
| **Covered face** refers to covering of mouth or nose while sneezing/cough/speaking loudly. *[Source: Krishnadas Bhattacharyya ,Rama Ram, SP Mitra,SK Bhattacharyya, TK Sarkar, U Dasgupta, DN Goswami. perceptions and practices of sputum positive pulmonary tuberculosis patients regarding their disease and its management. NTI Bulletin 2005,41/1&2, 11 – 17]* |
| **Adherence to treatment** means patients who adhere to TB medication as prescribed 95% of the time *[Niyi Awofeso. Anti-tuberculosis medication side-effects constitute major factor for poor adherence to tuberculosis treatment. Bull World Health Organ. 2008 Mar;86(3):B-D.]* |
| **Interrupted treatment** is defined as Patients who discontinued medication for 2-8 consecutive weeks but eventually restarted treatment were considered to have interrupted treatment*[World Health Organization. Treatment of tuberculosis: guidelines for national programmes.*  *Geneva, Switzerland: World Health Organization, 1997.]****.*** |
| **Interrupted treatment** refers to a patient whose treatment was interrupted for 2 consecutive months or more [Source: WHO. Global tuberculosis report 2012]. |
| **Follow-up spu­tum microscopy** refers patients should come to take spu­tum check at the end of 2, 5 and 6 month of treatment*[Tuberculosis Coalition for Technical Assistance. International Standards for Tuberculosis Care (ISTC). The Hague: Tuberculosis Coalition for Technical Assistance, 2006].* |
| **Abstained from smoking** refers to TB patients stop smoking during treatment of TB |
| **Abstained from alcohol** **drinking** refers to TB patients stop alcohol drinking during treatment of TB |
| **Nutrition improvement** refers to altering protein metabolism, micronutrient  deficiency (such as Vitamins A, D, E, C; minerals zinc, selenium) and anemia *[Source: Papathakis P, Piwoz E. Nutrition and Tuberculosis: A review of the literature and considerations for TB control programs. USAID/AED Africa's Health in 2010. Washington*  *DC, USA, 2008.]* |
| **Abstained from spitting** refers to stopping spitting in public places |
| **Safe method of sputum disposal** refers to avoiding indiscriminate spitting, disposing in dustbin, Burning or pouring boiled water in the container *[Source: Krishnadas Bhattacharyya ,Rama Ram, SP Mitra,SK Bhattacharyya, TK Sarkar, U Dasgupta, DN Goswami. perceptions and practices of sputum positive pulmonary tuberculosis patients regarding their disease and its management. NTI Bulletin 2005,41/1&2, 11 – 17]* |
| Reduce times to the public places |
| **A contact** is any person identified by a patient as a close contact during the course of the routine contact investigation *[Source: Supplemental interview for places of social aggregation. Centers for Disease Control and Prevention and Tuberculosis Epidemiologic Studies Consortium.]* |
| **Room Ventilation** refers to ventilating living room through negative pressure ventilation*[Source: WHO Guidelines for the Prevention of Tuberculosis in Health Care Facilities in Resource-Limited Settings]* |
